# Systemic but No Local Effects of Combined Zoledronate and Parathyroid Hormone Treatment in Experimental Autoimmune Arthritis

**DOI:** 10.1371/journal.pone.0092359

**Published:** 2014-03-17

**Authors:** Kresten Krarup Keller, Jesper Skovhus Thomsen, Kristian Stengaard-Pedersen, Ellen-Margrethe Hauge

**Affiliations:** 1 Department of Rheumatology, Aarhus University Hospital, Aarhus, Denmark; 2 Department of Biomedicine – Anatomy, Aarhus University, Aarhus, Denmark; Faculté de médecine de Nantes, France

## Abstract

**Introduction:**

Local bone erosions and osteoporosis in rheumatoid arthritis (RA) are the result of a more pronounced bone resorption than bone formation. Present treatment strategies for RA inhibit inflammation, but do not directly target bone erosions. The aim of the study was in experimental arthritis to investigate the juxtaarticular and systemic effects of simultaneous osteoclast inhibition with zoledronate (ZLN) and osteoblast stimulation with parathyroid hormone (PTH).

**Methods:**

Arthritis was induced in 36 SKG mice. The mice were randomized to three treatment groups and an untreated group: ZLN, PTH, PTH+ZLN, and untreated. Arthritis score and ankle width measurements were performed. Histological sections were cut from the right hind paw, and design-based stereological estimators were used to quantify histological variables of bone volume and bone formation and resorption. The femora were DXA- and μCT-scanned, and the bone strength was determined at the femoral neck and mid-diaphysis.

**Results:**

Locally, we found no differences in arthritis score or ankle width throughout the study. Similarly, none of the treatments inhibited bone erosions or stimulated bone formation in the paw. Systemically, all treatments improved bone mineral density, strength of the femoral neck and mid-diaphysis, and μCT parameters of both cortical and trabecular bone. In addition, there was an additive effect of combination treatment compared with single treatments for most trabecular parameters including bone mineral density and bone volume fraction.

**Conclusions:**

No local effect on bone was found by the combined action of inhibiting bone resorption and stimulating bone formation. However, a clear systemic effect of the combination treatment was demonstrated.

## Introduction

It is well known that patients with rheumatoid arthritis (RA) develop both local bone erosions and systemic osteoporosis. Local bone erosion in RA is mediated through bone resorption by the osteoclast at the cartilage-pannus junction [1]. Studies indicate that the bone formation is less pronounced in RA compared to the bone resorption [2], [3], and impact of RA on the Wnt signaling pathway is probably crucial to this process [4]. However, healing of erosions can be seen, but most often in patients with remission [5]. Osteoporosis in RA is mainly mediated by inflammation [6], resulting in an increased fracture risk independently of glucocorticoid use [7]. Treatment of RA has improved considerably over the last decade, but even with the best current therapies, a number of patients still have progression of erosive bone changes in the joints [8]. Present treatment strategies for RA inhibit inflammation, but do not directly target bone erosions. Consequently, treatment strategies that directly target bone are relevant.

Bisphosphonates are inhibitors of bone resorption, which are mainly used for treatment of osteoporosis [9]. The most powerful bisphosphonate is zoledronate (ZLN), and the effect on osteoporosis is well documented [10]. Parathyroid hormone (PTH) given intermittently is a powerful bone anabolic drug, which stimulates bone formation through the activation and maturation of cells of the osteoblast-line. Treatment with intermittent PTH increases BMD and decreases fracture risk in osteoporosis [11]. Inhibiting bone resorption with ZLN combined with stimulation of bone formation with PTH may inhibit development of erosions in the joints of RA patients, and in addition the combination may heal erosions. In clinical or experimental arthritis, no study has yet investigated the combined effect of ZLN and PTH.

The SKG mouse model for RA is characterized by symmetrical affection of peripheral joints (ankle, tarsal, and finger joints in all four limbs), rheumatoid factor, elevated cytokines (IL-1, Il-6, TNF-α, and Il-17), and systemic manifestations such as inflammation of the lungs and skin [12]–[15]. This model is also characterized by both local and systemic bone loss [16]–[18], and recently we demonstrated that systemic bone loss is present early after arthritis induction [19]. Consequently, the SKG model is suitable for studying the effect of bone targeting therapies in experimental chronic arthritis.

We hypothesize that stimulation of bone formation and inhibition of bone resorption may represent an effective future treatment strategy, for both local and systemic bone loss in RA. Hence, the objective of the present study was to investigate the juxtaarticular and systemic effects of simultaneous osteoclast inhibition with ZLN and osteoblast stimulation with PTH in the SKG mouse model of RA.

## Methods

### Animals, arthritis induction, and assessment of arthritis

The study comprised 36 8- to 10-week-old female SKG mice housed as previously described in detail [18]. Arthritis was induced with an intraperitoneal (i.p.) injection of 20 mg mannan suspended in 0.2 ml PBS, which induces the arthritis through the complement system [20].

Arthritis was scored twice weekly in accordance to the SKG scale [12]. In addition, the hind limb ankle width was measured every other week with an electronic sliding caliper, and the mean width of the right and left ankle joint was calculated. Both evaluations were performed by an observer blinded for the group distribution.

Calcein (Sigma-Aldrich, St Louis, MO, USA) 15 mg/kg and tetracycline (Sigma-Aldrich) 30 mg/kg fluorochrome labels was administered i.p. 8 and 4 days before the end of the study respectively. Eight weeks after arthritis induction (i.e. when the mice were 16- to 18-week-old), the mice were anesthetized with isoflurane (IsoFlo vet, Abbot Laboratories Ltd. Kent, UK) and euthanized by cervical dislocation.

### Ethics statement

The principles of laboratory animal care recommended by the US National Institute of Health were followed. The study was approved by the Danish Animal Experiments Inspectorate (permit number 2011/561-64).

### Treatment

Treatment was initiated 11 days after arthritis induction with mannan. The mice were assigned to 4 treatment groups (*n* = 9 per group): placebo+placebo (untreated), ZLN+placebo (ZLN), placebo+PTH (PTH), and ZLN+PTH. Human PTH (1–34) (Bachem, Bubendorf, Switzerland) or placebo were dissolved in saline and 2% mouse serum and subsequently injected subcutaneously (s.c.) at 80 μg/kg 5 times a week for 6 weeks and 3 days [21]. ZLN (Novartis, Basel, Switzerland) or placebo were injected s.c. at 100 μg/kg 5 times a week for 6 weeks and 3 days [22]. One mouse in the ZLN group had to be euthanized after 7 weeks due to a misplaced injection.

### Histological preparation of paws

After euthanization the right hind paw was cut 0.5 cm above the ankle joint and across the middle of the metatarsal bones, fixed in 70% ethanol, and subsequently embedded undecalcified in methylmethacrylate [23]. Tissue blocks were rotated randomly around a vertical axis through the longitudinal axis of the paw and 7-μm-thick sections were cut exhaustively on a microtome (Reichert Jung GmbH, Heidelberg, Germany) using the principles of vertical sectioning [24]. Approximately 10 levels each comprising 12 sections were acquired for each paw using the principles of systematic, uniform random sampling [25]. Finally, the sections were stained with Masson-Goldner trichrome, for tartrate-resistant acid phosphatase (TRAP), or left unstained for epifluorescent microscopy.

### Evaluation of histological sections

The microscope (Nikon ECLIPSE 80i, Tokyo, Japan) was equipped with a motorized Proscan 11 stage (Prior, Cambridge, UK), a MT1201 microcator (Heidenhain, Traunreit, Germany), a DP72 digital camera (Olympus, Tokyo, Japan), and a fluorescence illuminator (Prior, Cambridge, UK). The microscope system was connected to a PC with the stereological software newCAST (version 4.2.1.0, Visiopharm, Hørsholm, Denmark).

All parameters were evaluated in the tarsus at a total magnification of ×457. The tarsus was defined as all tarsal bones excluding the tibial and the metatarsal bones. Absolute bone volume (BV) was estimated using a point grid with an area per point of 36712 μm^2^. For absolute mineralizing surfaces (MS) and osteoclast-covered bone surfaces (Oc.S) a line grid with an area per length of 29.9 μm was used. These parameters were estimated according to stereological estimators as previously described in detail [18].

The mineralizing surfaces were defined as the number of intercepts with fluorochrome double labels plus half the number of intercepts with single labels. Mineral apposition rate (MAR) and bone formation rate (BFR) were estimated using the principles described by Dempster et al. [26]. Briefly, MAR was calculated as the mean of the interlabel width (iL.Wi) multiplied by the correction coefficient (π/4) and divided by the interlabel time. When double labels were absent a MAR value of 0.3 μm/day was used [27]. Subsequently, BFR was estimated as MS multiplied by MAR.

The average coefficient of error (CE) for the bone volume estimates was determined as earlier described to 2.5% [18]. CE for surface parameters could not be estimated, because the formula for CE of a surface is not valid due to the large variation in size between different sections of a mouse paw.

### Dual Energy X-ray Absorptiometry

After euthanization the right femora was exarticulated at the hip and the knee joint and stored in Ringer's solution at −20°C. The femora were thawed, dissected free of soft connective tissue, and placed in a standardized way in a peripheral DXA scanner (Sabre XL, Norland Stratec, Pforzheim, Germany) and scanned with a pixel size of 0.1 mm ×0.1 mm at a velocity of 2 mm/s. The areal bone mineral density (aBMD) was determined using the software provided with the scanner.

### Mechanical testing

After the DXA-scans, the maximum load of the mid-femoral diaphysis was determined by three-point bending test in a materials-testing machine (5566; Instron, High Wycombe, UK) as previously described in detail [19]. After the three-points bending test the proximal half of the femur was placed in a custom-made device for standardized fixation [28]. The fixation device was placed in the materials testing machine and compressive load was placed on the top of the femoral head at a constant velocity of 2 mm/min until fracture of the femoral neck. The measured data included the maximum load (F_max_) given in Newton (N).

### Micro Computed Tomography (μCT)

Before the three-point bending test, the cortical bone of the femoral mid-diaphysis was scanned in a desktop μCT scanner (Scanco μCT 35, Scanco Medical AG, Brüttiselen, Switzerland). A 0.82 mm high volume of interest (VOI) was scanned in high resolution mode (1000 projections/180°) with a spatial resolution of 3.5×3.5×3.5 μm^3^, an X-ray tube voltage of 55 kVp and current of 145 μA, and an integration time of 800 ms. The data sets were low-pass filtered using a Gaussian filter (σ = 0.8, support  = 1) and segmented with a fixed threshold filter (618.0 mgHA/cm^3^). The threshold was found as the midpoint between the peaks representing bone and marrow in the attenuation histogram [29]. The structural evaluation was performed with the software provided with the scanner (IPL, version 5.11, Scanco Medical AG, Brüttisellen, Switzerland). The measured data included: Cortical bone area (Ct.Ar) and average cortical thickness (Ct.Th).

After the three-point bending test, the distal half of the femur was placed in the μCT scanner and the distal femur was scanned with the same scanning parameters as the femoral mid-diaphysis. A 700-μm-high VOI was demarcated in the distal femoral metaphysis starting 950 μm beneath the lowest point of the hyaline cartilage in the epiphyseal growth plate thereby excluding the primary spongiosa from the analysis. The data sets were low-pass filtered using a Gaussian filter (σ = 1.3, support  = 2) and segmented with a fixed threshold filter (589.6 mgHA/cm^3^). The threshold was found in the same way as for cortical bone. Measured data included: volumetric bone mineral density (vBMD), trabecular thickness (Tb.Th*), trabecular separation (Tb.Sp*), trabecular number (Tb.N*), structure model index (SMI), and bone volume fraction (BV/TV) [29], [30].

### Statistics

Data were analyzed using STATA (version 11, Statacorp, College station, USA). Groups were compared with one-way ANOVA. Normality was checked with QQ-plots. If the hypothesis of equal means in the groups were rejected, pair wise comparisons between groups were made. All treatment groups were compared with untreated animals and if the hypothesis of no difference was rejected it was tested whether the hypothesis of no difference between combination treatment and the two other treatment groups could be rejected. If data were not normally distributed, Kruskal-Wallis test was performed, followed by the two sample Wilcoxon rank-sum test (Mann Whitney's U test). P-values below 0.05 were considered statistically significant.

## Results

### Arthritis score and ankle width

**Figure 1:**
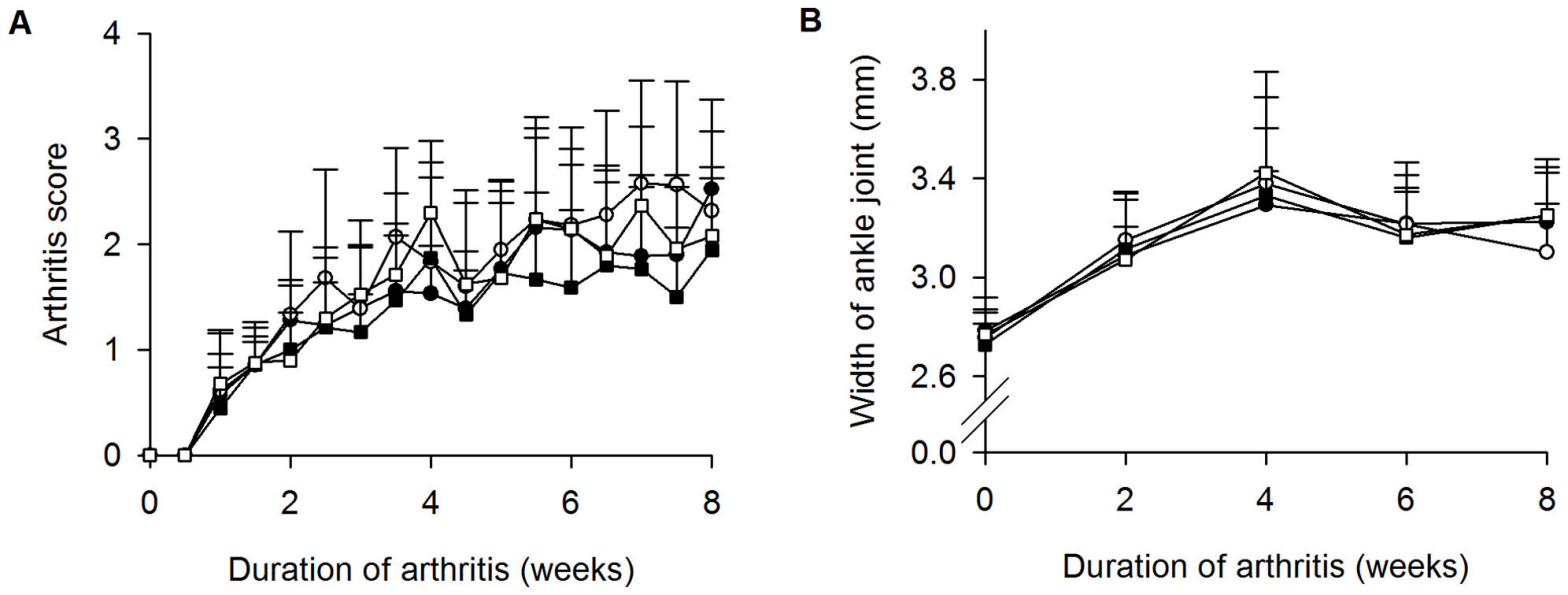
Similar severity of arthritis in the different groups. Arthritis score (**A**) and mean width of hind limb ankle joints (**B**). Untreated (□), PTH (▪), ZLN (○), and ZLN+PTH (•). Values are mean plus standard deviations. N = 9 mice per group.

Arthritis score was similar for all groups throughout the study (Figure 1A). Likewise, the mean width of the hind limb ankle increased in all groups with time, but no differences were detected between the groups at any time point throughout the study (Figure 1B).

### Histological evaluation of bone in mouse paws

**Figure 2:**
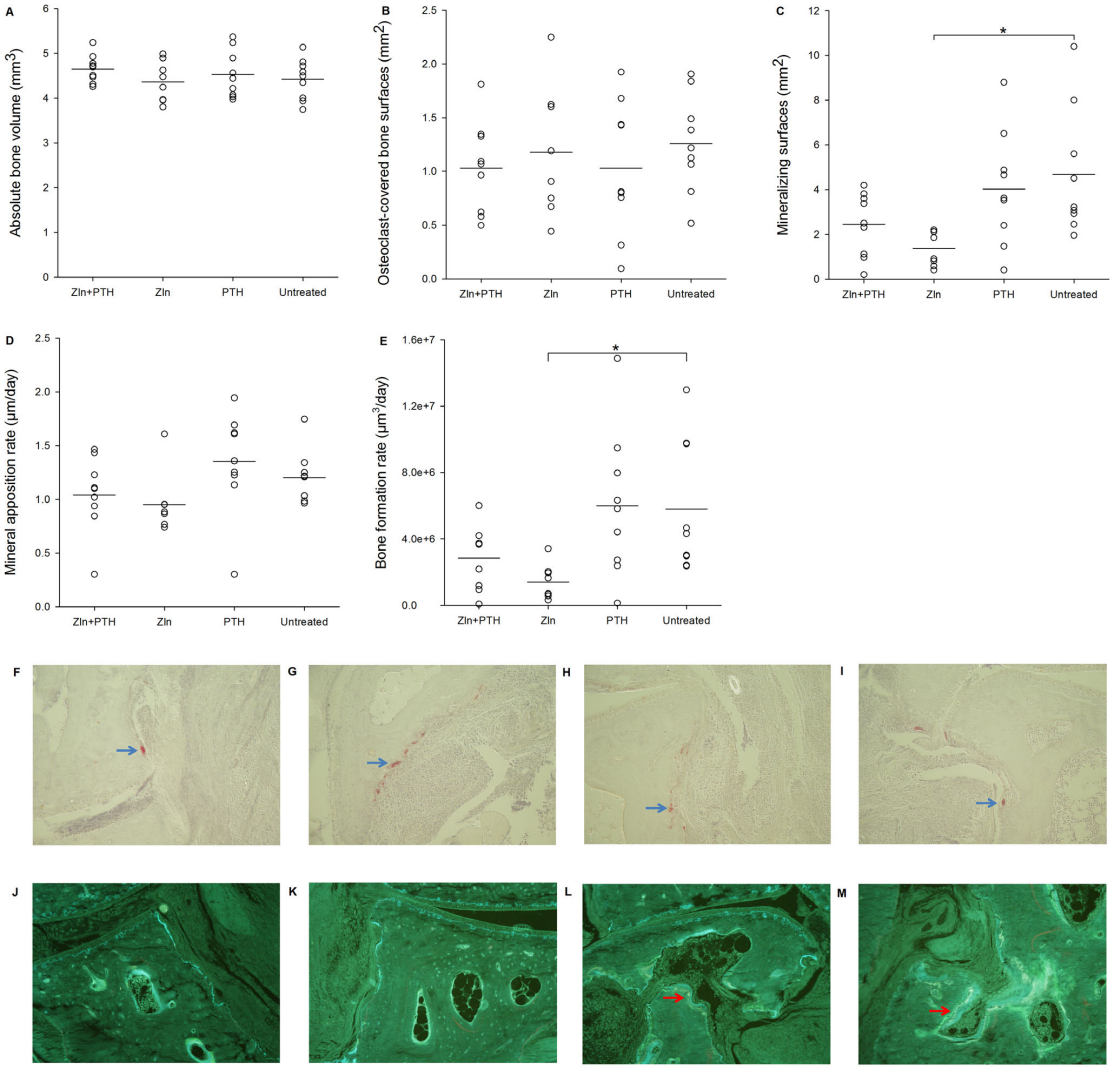
Bone changes in mouse paws evaluated with stereology of the right hind paw. Horizontal lines indicate mean values. N = 8–9 mice per group and *  = *p*<0.01. Representative pictures of are demonstrated (total magnification ×457): PTH+ZLN (**F and J**), ZLN (**G and K**), PTH (**H and L**) and Untreated (**I and M**). Red cells indicate osteoclasts in the TRAP stained sections (blue arrows) (**F–I**). Tetracycline (yellow) and calcein (green) labels are demonstrated in unstained sections (red arrows) (**J–M**).

The BV of the tarsus, evaluated with stereological estimators, did not differ between the groups (Figure 2A). Likewise, Oc.S did not differ between the groups indicating similar bone resorption in the different groups (Figure 2B). MS was significantly lower in the ZLN group than in the untreated group (*p*<0.01) (Figure 2C). Furthermore, MAR did not differ between the groups, whereas BFR was significantly lower in the ZLN group than in the untreated group (*p*<0.01) (Figure 2 D&E). Representative pictures of histological sections are supplied in Figure 2.

### DXA and mechanical testing of the femora

**Figure 3:**
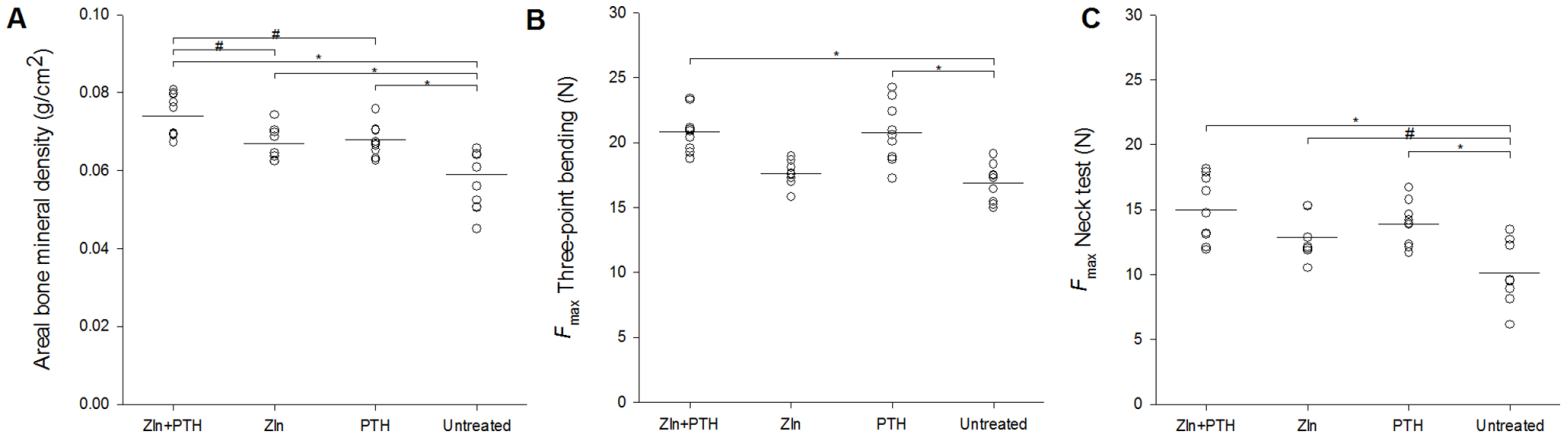
Area bone mineral density evaluated by DEXA and bone strength of the right femur. Area bone mineral density of the total femur in the different groups (**A**). Bone strength was measured in the mid-diaphysis with three-point bending (**B**) and in the femoral neck with compression testing (**C**). N = 7–9 mice per group. *  = *p*<0.01 and #  = *p*<0.05.

The aBMD of the total femur was significantly higher in all treatment groups than in the untreated group (*p*<0.01). In addition, the aBMD was significantly higher in the PTH+ZLN group than in both the ZLN and the PTH group (*p*<0.05) (Figure 3A).

The fracture strength of the femoral mid-diaphysis was significantly higher in the groups treated with PTH than in the untreated group (*p*<0.01) (Figure 3B). In addition, the femoral neck was significantly stronger in all treatment groups than in the untreated group (*p*<0.01 and *p*<0.05). However, at the femoral neck the combination treatment did not result in significantly stronger bone than the single treatments (Figure 3C).

### μCT of femoral trabecular bone

**Figure 4:**
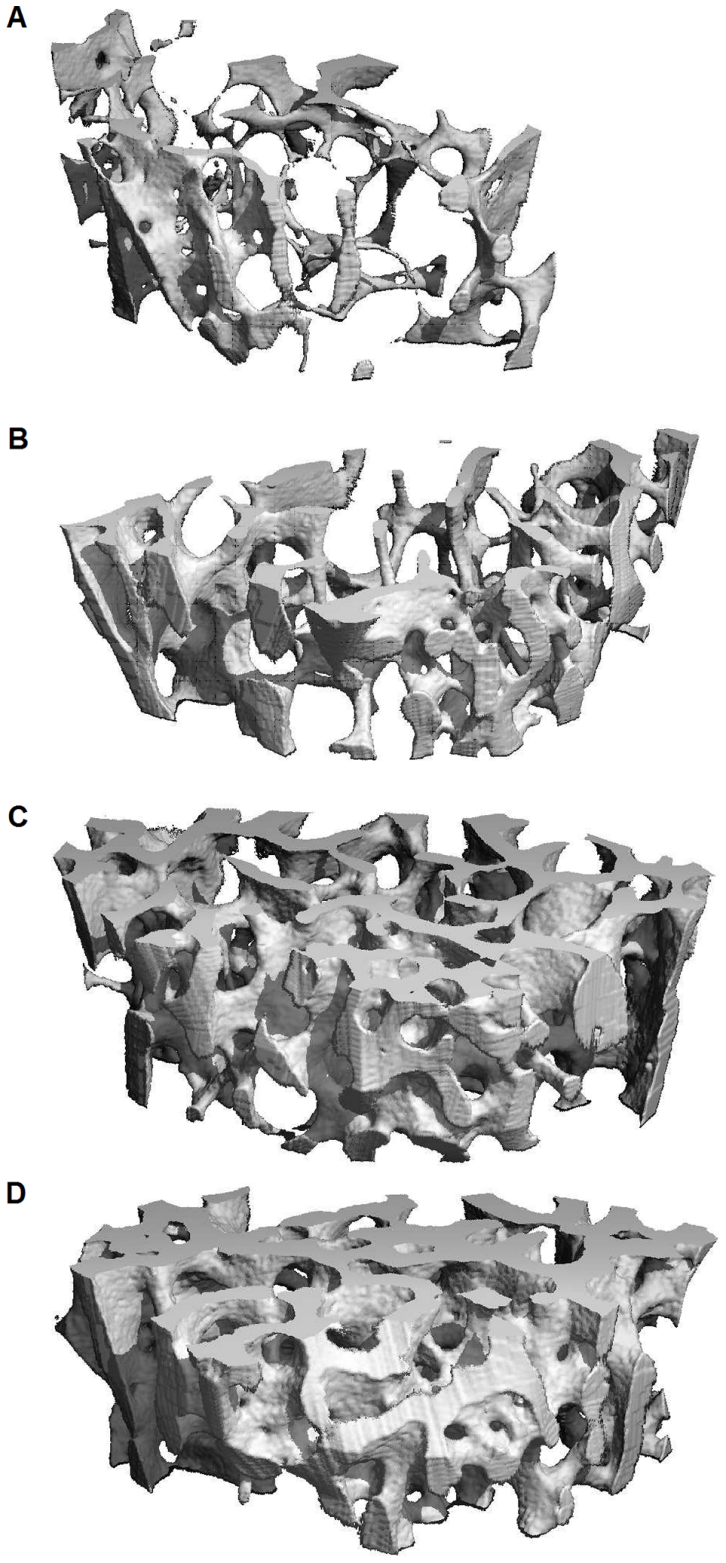
μCT pictures of trabecular bone. Representative μCT pictures of trabecular bone in the femur metaphysis. Untreated (**A**), PTH (**B**), ZLN (**C**) and PTH+ZLN (**D**) treated mice are demonstrated.

**Figure 5:**
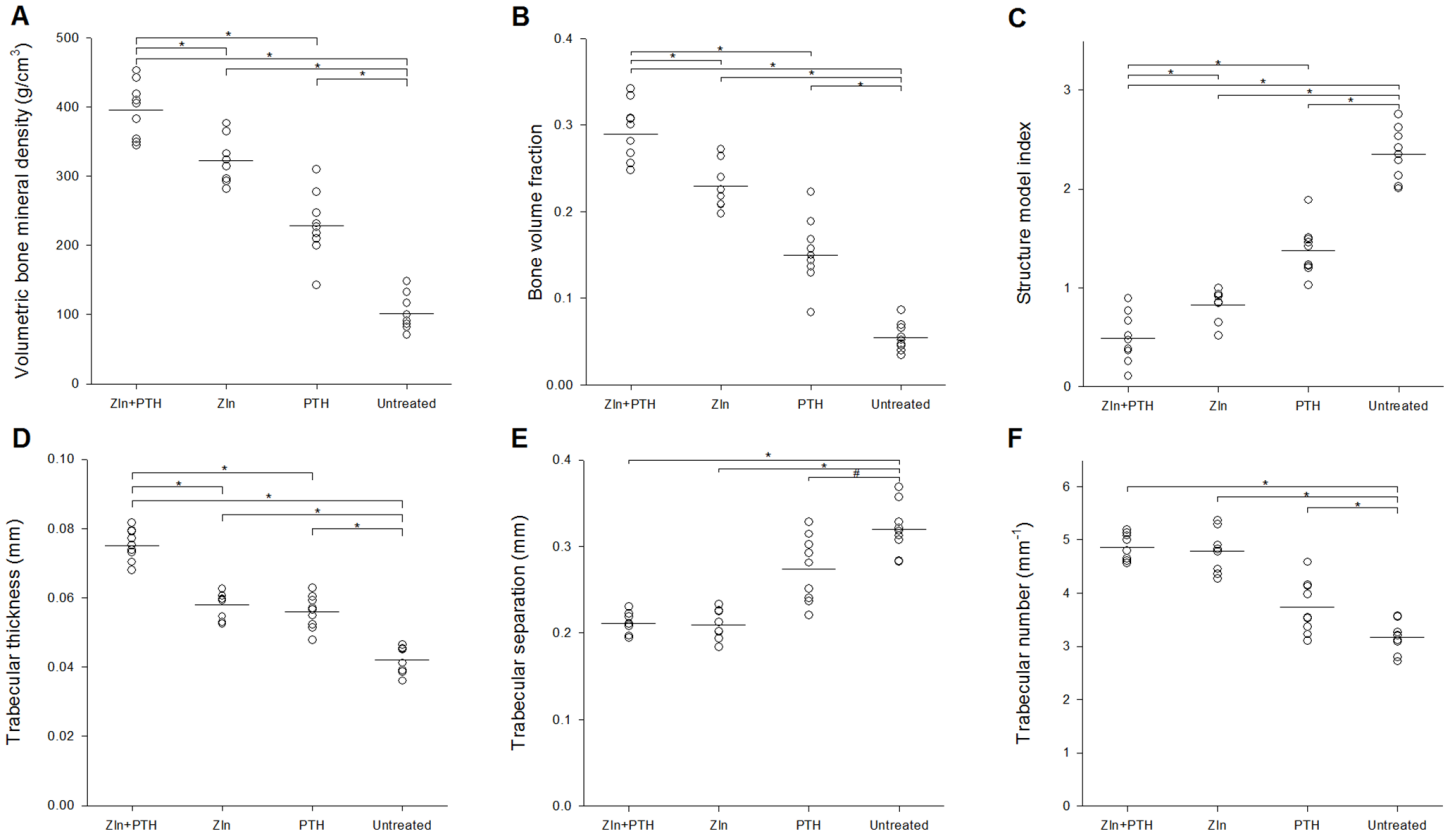
μCT parameters from trabecular bone. Results from μCT evaluation of trabecular bone in the femoral metaphysis of the right femora. N = 8–9 mice per group. *  = *p*<0.01 and #  = *p*<0.05.

Trabecular bone was evaluated in the distal femoral metaphysis, and as illustrated in Figure 4, substantial differences between groups were demonstrated. The vBMD of the distal femoral metaphysis was significantly higher for all treatment groups than for the untreated group. In addition, the vBMD for the combination group was significantly greater than for the two single treatment groups (*p*<0.01) (Figure 5A). The results for BV/TV were similar (Figure 5B). SMI was significantly lower in the treatment groups than in the untreated group indicating that the trabeculae in the treated animals were more plate-like. Moreover, there was an additive effect of combination treatment compared to single treatments (*p*<0.01) (Figure 5 C). Trabecular thickness Tb.Th* was higher in the treatment groups than in the untreated group, and in addition Tb.Th* was higher in the combination group than in the two single treatment groups (*p*<0.01) (Figure 5D). Finally, Tb.Sp* was lower and Tb.N* was higher in the treatment groups than in the untreated group (*p*<0.01 and *p*<0.05) (Figure 5E&F).

### μCT of femoral cortical bone

**Figure 6:**
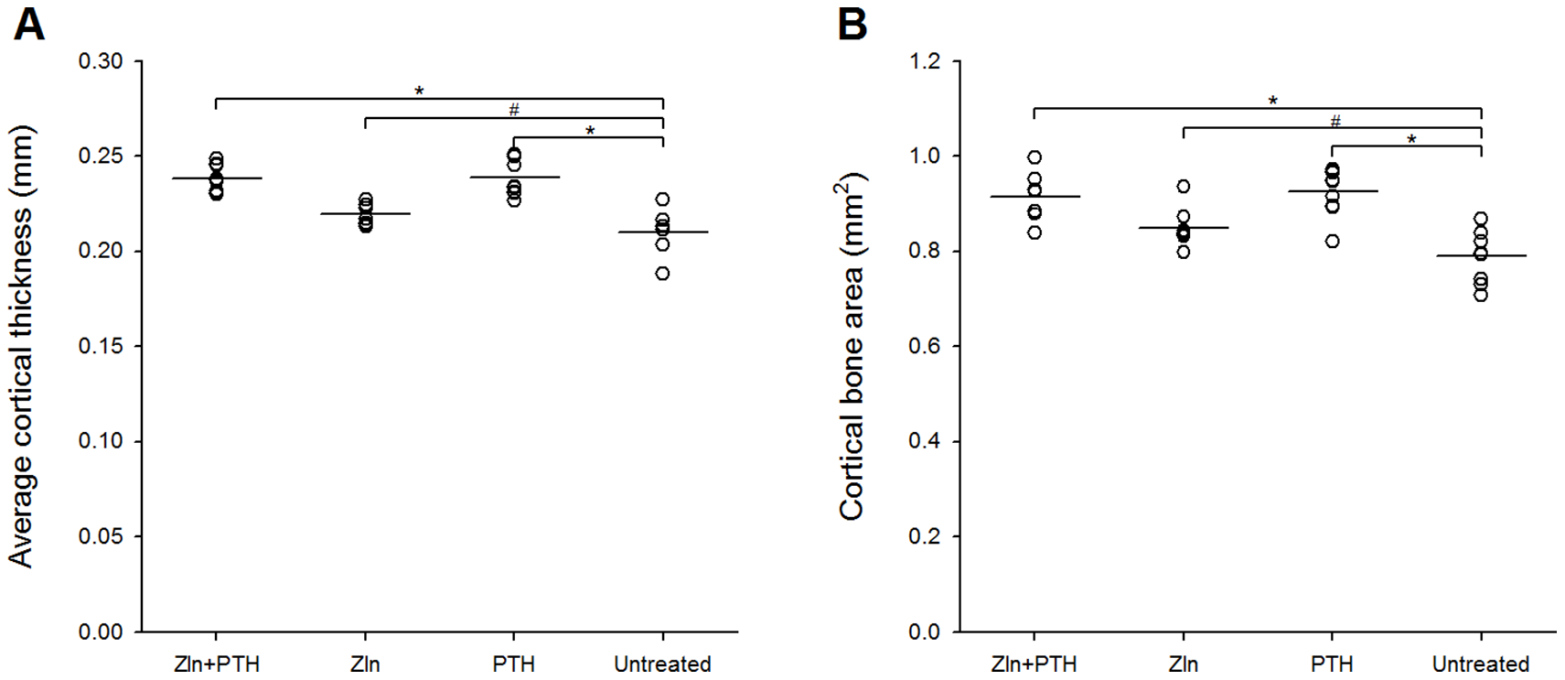
μCT parameters from cortical bone. Results from μCT evaluation of cortical bone in the mid-diaphysis of the femur. N = 8–9 mice per group. *  = *p*<0.01 and #  = *p*<0.05.

Cortical thickness Ct.Th at the femoral mid-diaphysis was greater in all treatment groups than in the untreated group, but there was no additive effect of the combination treatment (*p*<0.01 or *p*<0.05) (Figure 6A). Likewise, the cross sectional cortical bone area Ct.Ar was higher in the treatment groups than in the untreated group (*p*<0.01 or *p*<0.05) (Figure 6B).

## Discussion

During the last decades, treatment of RA has improved considerably, but local bone erosions and osteoporosis are not prevented in all patients. Currently, treatment is mainly focused on inhibition of the inflammation, whereas bone loss is not directly targeted. In the present study we investigated the combined effect of the bone anti-resorptive zoledronate (ZLN) treatment and the bone anabolic intermittent parathyroid hormone (PTH) treatment.

The systemic effect of ZLN for osteoporosis is well known and documented [10]. Previous experimental [22], [31] and clinical studies [32] have found that ZLN is also an effective drug against inflammation-induced bone loss in RA. A finding, which has been confirmed by the present study, as we showed that femoral neck fracture strength, femoral aBMD, and femoral cortical and trabecular μCT parameters improved compared with the untreated group. In contrast, there was no improvement of the fracture strength of the femoral mid-diaphysis, which is probably due to the lack of remodeling in cortical bone in rodents.

In the present study ZLN did not prevent the development of local bone erosions. Bone volume and resorption were unchanged, and actually a suppression of bone formation was seen. This is at variance with previous animal studies, where a clear preventive effect of ZLN on bone erosions was demonstrated [22], [31], [33]. There may be several explanations for the conflicting results. In the studies various animal models were used. The SKG model results in a chronic RA-like arthritis, whereas e.g. the collagen arthritis model used in some of the other studies are more acute and burns out after a short time. In addition, in the present study we performed stereology, whereas histomorphometry was performed in the previous studies. Stereology based quantitative histology is free of assumptions concerning shape of the tissue and orientation, and it gives quantities in absolute volume, area, length, or numbers. On the other hand conventional histomorphometry is based on assumptions of shape and orientation and reports data in 2D-terms such as area and perimeter. Finally, the dose of ZLN was not exactly the same in all studies. ZLN has been used in one randomized controlled trial with RA patients [32]. In that study no difference between the treatment groups were found in the primary endpoint of erosions visualized by magnetic resonance imaging (MRI). In general, human studies of RA treatment with bisphosphonates have been disappointing concerning the effect on joint erosions [34], [35]. A reason may be that ZLN inhibit osteoclasts directly, but do not inhibit the recruitment of osteoclasts through the receptor activator of nuclear factor κB ligand (RANKL) [36]. It is conceivable that the inflammatory signal for osteoclastogenesis locally in the joint is so powerful that it can outweigh the osteoclasts killed by the ZLN treatment. Blocking of the osteoclast recruitment may therefore inhibit local bone erosions. This view is supported by a clinical study of the RANKL inhibitor denosumab in RA, which was able to arrest the progression of erosions as assessed by both MRI and conventional radiographs [37]. Consequently, it would be interesting to study inhibition of both the inflammation and the osteoclasts in a future study. In summary, our results illustrates that ZLN may not effectively inhibit osteoclastic resorption in joints with arthritis, and thus not inhibit local bone erosions. Moreover, ZLN may be potentially harmful, because bone formation is further inhibited, which could hamper the healing of existing joint erosions in RA.

Intermittent PTH is an effective bone anabolic treatment regimen for postmenopausal osteoporosis [11]. We evaluated the systemic effect of intermittent PTH on inflammation-induced bone loss, and found a clear effect. Femoral neck and mid-diaphyseal bone strength, aBMD, and cortical as well as trabecular μCT parameters improved compared with the untreated group. Our study confirms the experimental results published by others [21], [38]. However, the effect in clinical trials with RA patients remains to be seen.

We were not able to demonstrate an effect of PTH locally in the joint, and this result is consistent with other studies. One group found no local effect of PTH in collagen-induced arthritis (CIA) in rats [38]. Another group found no effect of PTH alone on bone erosion in the TNF-transgenic mouse model, whereas a combination with anti-TNF was effective [21]. Furthermore, the same researchers demonstrated no effect on local erosions using the CIA and the adjuvant-induced arthritis model in rats, but found a clear effect if PTH was combined with either inhibition of RANKL or TNF [39]. These results indicate that directly or indirectly inhibition of osteoclast differentiation and maturation may be important for the local effect of PTH in arthritis. Therefore, PTH may potentially heal bone erosions in RA, but should be combined with inhibition of osteoclast differentiation.

In the present study the main objective was to investigate the combined effect of osteoblast stimulation by PTH and osteoclast inhibition by ZLN. The combination therapy was not effective on the inflammation as ascertained by arthritis score. This finding was expected because neither of the two single therapies improved inflammation. In fact, ZLN may even enhance inflammation [33], although we did not find evidence for this in our study. We also investigated the effect of combination therapy on local bone changes and were only able to demonstrate an inhibition of bone formation. RA is characterized by an increased osteoclastic bone resorption and an inhibition of bone formation, and we hypothesized that targeting both, would reduce the presence of bone erosions. However, as discussed above, ZLN inhibited bone formation and a potential positive effect of PTH on bone formation was simply not able to reverse this inhibition. In contrast, there was an effect of the combination treatment on systemic bone loss. Thus, we found an additive effect of the combination therapy, which improved trabecular bone structure, but did not translate into an additive effect on bone strength. There was no additional effect of combination therapy on cortical parameters. Again, the reason may be the lack of effect of ZLN, because of absent cortical bone remodeling in rodents. The effect of stimulating bone formation and inhibiting bone erosions varied locally and systemically in our study. Thus, the mechanisms for inducing local and systemic bone loss are probably very different and the strong inflammatory drive on the local bone resorption and formation compared with the systemic bone remodelling is probably crucial. The local bone loss is influenced by local cytokines in the joint such as TNF-alpha and IL-1 modulating Wnt signalling in osteoblasts leading to inhibited bone formation as well as stimulated bone resorption through RANKL [4], [40], [41]. Synovial fibroblasts and local glucocorticoid synthesis may play a role in the different local bone response to inflammation [42].

Our study is the first to investigate and demonstrate the combined effect of PTH and a bisphosphonate in arthritis. In clinical osteoporosis research the effect of this combination is still under debate. One research group did not find an effect of combining alendronate and PTH in a clinical trial [43]. Another group found an effect on BMD if PTH was added to alendronate treatment [44] and an early increase in BMD in combination treatment with PTH and ZLN compared to single treatment [45]. Recently a significant effect was demonstrated by adding PTH to ibandronate [46].

There are limitations to our study. Systemic bone loss was investigated in the femur and not in the spine, which is a typical site for studying systemic bone loss. However, arthritis in SKG mice does not affect the knee joint [12]. Hence, the observed femoral bone changes are indeed caused by a systemic and not a local effect of arthritis.

In conclusion, we demonstrated that combination treatment with ZLN and PTH was effective on systemic but not on local bone loss in a mouse model of RA. Therefore, this combination treatment may represent an effective strategy to prevent osteoporosis in RA, whereas local bone erosions should be treated in a different manner, probably by targeting the osteoclast differentiation or combining osteoclast inhibition with inhibition of inflammation. Hence, different mechanisms may influence local and systemic bone loss in arthritis.
